# Using Immersive Environments in E-Mental Health Rehabilitation Programs Directed to Future Health Professionals to Promote Empathy and Health Literacy about Schizophrenia

**DOI:** 10.3390/healthcare12151550

**Published:** 2024-08-05

**Authors:** Paulo Veloso Gomes, António Marques, Javier Pereira, Rui Pimenta, João Donga, Raquel Simões de Almeida

**Affiliations:** 1 Laboratório de Reabilitação Psicossocial (LabRP), Centro de Investigação em Reabilitação (CIR), Escola Superior de Saúde (ESS), Polytechnic of Porto, 4200-072 Porto, Portugal; ajmarques@ess.ipp.pt (A.M.); afa@ess.ipp.pt (R.S.d.A.); 2 CITIC Research Center, University of A Coruña, 15011 A Coruña, Spain; javier.pereira@udc.es; 3 Associated Laboratory for Green Chemistry of the Network of Chemistry and Technology (LAQV-REQUIMTE), Escola Superior de Saúde (ESS), Polytechnic of Porto, 4200-072 Porto, Portugal; rep@ess.ipp.pt; 4 Center for Innovation in Biomedicine and Biotechnology (CIBB), Centro de Estudos de Investigação em Saúde da Universidade de Coimbra (CEISUC), 3004-512 Coimbra, Portugal; 5 Laboratório de Reabilitação Psicossocial (LabRP), Centro de Investigação em Reabilitação (CIR), Escola Superior de Media Artes e Design (ESMAD), Polytechnic of Porto, 4200-072 Porto, Portugal; jpd@esmad.ipp.pt

**Keywords:** e-mental health, empathy, health literacy, schizophrenia, rehabilitation, virtual reality, 360° video, immersive environments

## Abstract

Rehabilitation involves all types of patients, including people with schizophrenia. Schizophrenia is considered a complex syndrome characterized in general by fundamental and characteristic distortions of thinking and perception. The quality of life of a person with schizophrenia can be compromised by difficulty in carrying out their daily tasks and by the social stigma of their condition. The importance of training and sensitizing students in rehabilitation areas to this type of problem to improve the rehabilitation processes in which they will participate as future professionals involves empathy and the ability to communicate with these populations. It is possible through virtual reality to create immersive environments to simulate some psychotic symptoms characteristic of people with schizophrenia, such as visual hallucinations and hearing voices. The aim of this study was to test the effect of exposure to experiences characteristic of schizophrenia through two different types of immersive environments, graphical computational virtual reality and 360° video, on students from areas of social rehabilitation regarding empathy, social distance, and attitudes towards people with schizophrenia. Although the results were positive for the three parameters under study, no significant differences were found for each of them between the environments to which the participants were exposed. This study concluded that the choice between the two types of immersive environments should be based on the project’s objectives, the target audience’s needs, and available resources, rather than the type of environment itself, as their impact was similar.

## 1. Introduction

Schizophrenia is considered a complex syndrome characterized by fundamental and characteristic distortions of thinking and perception [1]. The quality of life of a person with schizophrenia can be compromised not only by the difficulty in carrying out their daily tasks, but also due to stigma. According to research conducted in 16 countries, the prevalence of perceived stigma among people with schizophrenia was found to be 13.5%—in developing nations, 22.1%, and in developed nations, 11.7% [2]. Another study that was conducted in 14 European nations found that 41.7% of schizophrenia patients experienced stigma [3], and this rate might be higher because many are unable to report their stigma due to their symptoms. Three distinct subtypes of stigma can be distinguished [4]—because of internalizing negative stereotypes, around 90% of patients with schizophrenia acquire internalized or self-stigma [5]; interpersonal or social stigma happens when the stigmatized person is socially rejected and isolated [4,6]; organizational actions that discriminate against stigmatized groups are what lead to institutional stigma [7]. Some authors also mention stigma by association, referred to as “courtesy stigma”, which occurs when the effects of stigma are extended to someone connected to a person with mental illness, such as family members [8].

In the literature, self-stigma was identified as a major barrier to recovery from schizophrenia and to psychosocial rehabilitation adherence [3,9]; nevertheless, mental health rehabilitation professionals’ stigma towards people with schizophrenia has been re-searched, and the results are contradictory, since in some studies, they are one of the main sources of stigmatization [10,11,12,13]. According to several epidemiologic studies, the kind and intensity of psychopathology have an impact on how common it is among people with schizophrenia to perceive stigma [14]. Several studies argue that having personal contact with people with mental illness has positive effects on reducing stigma towards them [15], and that is why future healthcare professionals should interact with patients with schizophrenia through internships [16] during professional training. However, that is not always possible. Additionally, evidence shows that empathy is negatively correlated with stigma [17,18]. Empathy is a complex emotional and cognitive response influencing human behavior and encompasses the ability of one person to share and understand the internal states of someone else [19]. Moreover, empathy, defined as the ability to understand and share the feelings of another, is a key facilitator of communication and the creation of humanized interactions [20]. In healthcare settings, developing empathy among professionals can significantly reduce stigma, as it fosters more understanding and acceptance of patients’ experiences, ultimately leading to higher satisfaction levels and better health outcomes [21,22].

Some studies have proposed the use of virtual reality-based programs for boosting empathy [23,24]. Augmented reality and virtual reality-based interventions give participants the chance to explore and engage with realistic environments, making them effective psychoeducational tools for mental illness [25]. Positive psychotic symptoms like delusions and hallucinations are complex and subjective experiences that are challenging to comprehend. One of the applications of virtual reality is precisely to simulate these difficult or intangible conditions so that students can experience it firsthand [26]. For that reason, these technologies are growing in popularity in healthcare and health profession education fields, as they provide students with the opportunity to immerse them-selves in realistic simulations, making them an effective teaching and learning tool [27,28]. Consequently, not only enhancing empathy, but also raising mental health literacy awareness is easier with immersive digital environments. Even using 360-degree videos of different scenarios replicating clinical symptoms of schizophrenia via head-mounted displays (HMDs) seems to be perceived as effective, useful, and exciting [29]. On the other side, simulated hallucinations can have contradictory effects on stigma, increasing empathy but also the desire for social distance [30].

Therefore, it is important to better understand how these novel technologies could be used effectively to train mental health rehabilitation professionals who are more capable of interacting and communicating with and rehabilitating people with schizophrenia. Thus, this study aims to compare the impact on empathy, social distance, and attitudes towards people with schizophrenia of two different types of immersive environments, graphical computational virtual reality and 360-degree video, on students in rehabilitation areas.

### 1.1. Mental Health Literacy

For many years, the construct of health literacy has been examined, and its significance in illness prevention and health promotion has been emphasized. Promoting health literacy among individuals, communities, and organizations has significant potential and challenges for public health. The term mental health literacy (MHL) originally appeared in a study conducted by Jorm and colleagues in 1997 [31]. MHL is defined by these authors as knowledge and beliefs regarding mental diseases that help in their recognition, management, and prevention. It includes (1) the recognition of disorders or types of psychological distress; (2) the knowledge and beliefs about risk factors and causes; (3) knowledge and beliefs about self-help interventions; (4) knowledge and beliefs of professional help available; (5) attitudes that facilitate recognition and appropriate help-seeking; and (6) knowledge of how to find mental health information [32]. Jorm later expanded the definition of MHL to encompass not only knowledge about mental illnesses but also the ability to apply this knowledge to improve one’s own health and the health of others [33]. This emphasis on actionable knowledge aligns with this study’s focus on utilizing immersive environments to enhance empathy and reduce stigma, highlighting the role of MHL in creating more supportive and informed healthcare interactions.

Empathy is regarded as an essential component of the patient–provider relationship in healthcare professions [34]. Empathy is crucial in the patient–health professional relationship, as it helps build trust, facilitates communication, and improves patient satisfaction. It allows healthcare providers to understand and respond to patients’ emotions, concerns, and needs, which can lead to better treatment adherence and overall health outcomes. Empathetic interactions can also reduce patient anxiety and contribute to a more compassionate and supportive healthcare experience [35]. The healthcare professional plays a crucial role in the rehabilitation process, as their sensitivity to each situation and empathy are paramount. These qualities enable emotional support to be tailored to each specific situation [36]. Given the importance of empathy and reducing stigma in clinical communication in rehabilitation, this study started from the research question: “What is the comparative impact of Graphical Computational Virtual Reality versus 360° Video immersive environments on empathy, social distance, and attitudes towards individuals with schizophrenia among students in social rehabilitation fields?”.

### 1.2. The Sense of Presence in Immersive Environments

Over the past twenty years, virtual reality (VR) has profoundly impacted the field of psychological research. This technology’s unique ability to replicate complex real-world scenarios has opened new opportunities for the controlled study of human behavior in laboratory settings. Since VR effectively simulates reality, it heavily relies on the careful selection of perceptual cues to evoke emotional responses. These emotional experiences are intrinsically linked to the concept of “presence”, which denotes a user’s profound sensation of genuinely inhabiting a virtual environment [37].

The sense of presence refers to the feeling of being fully immersed in a particular environment or situation, to the extent that one’s awareness of the physical world diminishes, at least in some cases. Whether experienced through virtual reality or in everyday life, the sense of presence holds profound implications for our understanding of consciousness, perception, and the human experience [38].

Media experiences such as cinema and VR can be so immersive and captivating that the observer loses some awareness of their physical surroundings and reacts physically and emotionally as if they were present in the mediated environment [39]. At its essence, the sense of presence centers on the notion of being “here” rather than “there”. It entails a deep shift in consciousness where one’s attention and awareness are primarily directed toward the present moment or a specific environment [39]. This phenomenon can manifest in various ways and is not confined to any specific sensory modality. It can be experienced visually, auditorily, kinesthetically, intellectually or even emotionally.

In the realm of technology, VR offers one of the most vivid examples of the sense of presence. VR immerses users in computer-generated environments, engaging multiple senses simultaneously to create a compelling illusion of presence. The combination of realistic visuals, spatial audio, and haptic feedback can transport individuals to entirely different worlds, making them feel as if they are physically present in those digital realms. This immersion goes beyond mere visual and auditory experiences; it extends to the perception of depth, scale, and physicality, further enhancing the sense of presence.

The sense of presence is rooted in cognitive and perceptual processes [40]. In VR, it is typically associated with the following:

- Perceptual Realism: The fidelity of sensory information in VR, including visual, auditory, and haptic feedback, greatly influences the sense of presence. High-quality graphics, spatial audio, and responsive haptic feedback contribute to a more immersive experience.
- Immersion: The extent to which participants can suspend disbelief and become fully engaged with the virtual environment is a crucial factor in generating presence. Immersion often entails a sense of agency, where participants feel in control of their actions within the virtual world.
- Spatial Cues: Accurate spatial mapping, tracking of head and body movements, and consistent feedback from the virtual environment contribute to participants’ sense of presence. These cues help create a coherent and convincing virtual experience.

The sense of presence in VR, the feeling of “being there” in a virtual environment, plays a pivotal role in eliciting emotions [41]. Emotions are fundamental to the human experience, and virtual reality has emerged as a powerful tool for eliciting, amplifying, and understanding these emotions. The sense of presence in VR, which immerses participants in a simulated environment, influences individuals’ emotional responses.

The sense of presence is closely intertwined with emotional experiences in VR. Key elements include the following:

- Immersion and Engagement: A heightened sense of presence can result in increased participant immersion and emotional engagement within the virtual environment. This enhanced immersion allows participants to emotionally connect with the content.
- Emotional Transfer: In VR, emotions can be transferred from the virtual world to the participant. This transfer is facilitated by the vivid sensory input, including visuals, audio, and haptic feedback, creating a sense of emotional contagion within the virtual space.
- Presence and Realism: The more realistic the virtual environment, the more it can evoke emotional responses. Realistic environments and characters are more likely to elicit emotions such as fear, joy, or empathy.

Emotions are a significant component of how presence is perceived. They influence all behaviors, cognitions, and conscious and unconscious interactions between the individual and the environment, thereby impacting presence [42].

Virtual reality is a versatile platform for eliciting a broad spectrum of emotions [43], including but not limited to:

- Fear and Anxiety: Horror games and simulations can evoke strong feelings of fear and anxiety, often heightened by a strong sense of presence. Participants may feel genuinely threatened, despite being aware that it is a virtual experience.
- Empathy and Compassion: VR can cultivate empathy by enabling participants to embody different characters or immerse themselves in scenarios that evoke compassion, such as witnessing the experiences of refugees or victims of natural disasters.
- Joy and Awe: VR can transport participants to breathtaking landscapes or provide surreal experiences, evoking feelings of joy, awe, and wonder.
- Discomfort and Nausea: While VR can evoke positive emotions, it can also cause discomfort, motion sickness, or anxiety in some individuals, affecting the sense of presence and emotional responses.

The relationship between the sense of presence that immersive environments provide and the creation of emotions and attitudes is crucial for understanding how VR and other immersive settings impact human experiences. Here are some ways in which these elements are interconnected:

- Interaction with the environment: The sense of presence in immersive environments arises from participants feeling like an integral part of the virtual environment. When individuals interact with this environment in a meaningful way, whether in games, simulations, or training, they experience emotions more intensely and authentically [44]. For example, in a virtual reality environment that simulates an amusement park, participants can experience excitement and joy similar to what they would feel in a real park.
- Empathy and emotional connection: In immersive environments, the sense of presence can be so strong that participants develop empathy with virtual characters or simulated situations. This can lead to the creation of emotions such as sympathy, compassion, or anger, depending on the narrative or interactions in the virtual environment [45]. For example, in a simulation of social injustice situations, participants may experience indignation, which can influence their real-world attitudes.
- Learning and therapeutic exposure: In training and therapy contexts, the sense of presence in immersive environments is often utilized to create controlled experiences that evoke desired emotions and attitudes. For example, in exposure therapy for post-traumatic stress disorder (PTSD), an immersive environment can assist patients in safely revisiting traumatic situations, thereby reducing associated anxiety and fear.
- Influence on attitudes and behaviors: The sense of presence experienced in immersive environments can impact participants attitudes and behaviors [46]. If someone undergoes a simulation highlighting the dangers of driving under the influence of alcohol, it can lead to a positive attitude change towards not driving under the influence, ultimately influencing their real-world behavior.

The sense of presence in immersive environments is closely linked to the creation of emotions and attitudes because these environments have the ability to deeply and authentically engage users. This makes them powerful tools for influencing human behavior, whether for therapeutic, educational, entertainment, or commercial purposes.

As interactivity is an important factor that contributes to the sense of presence increasing immersion, it is important that when comparing different types of environments, their degree of interactivity is similar.

## 2. Materials and Methods

### 2.1. Study Design

In this experimental study, the same outcome was measured before and after the exposure, two groups were defined, randomized and evaluated two times, following a pre- and post-test methodology [47].

### 2.2. Participants

The participants, students in rehabilitation areas, were recruited from the School of Health—Polytechnic of Porto, Portugal. These students will be future health professionals and will join rehabilitation teams.

A total of 100 students were selected for the study, comprising 20 men and 80 women. The sample was stratified by sex and then randomly divided into two groups, with each group consisting of 10 men and 40 women. Individuals were randomly assigned to either the control or experimental group within each gender category using a random-number table. This approach was employed to minimize selection bias and ensure that any observed differences between the groups could be attributed to the intervention rather than pre-existing differences, thereby maintaining the study’s internal validity. After conducting the data collection, 3 female participants from the control group and 9 female participants from the experimental group were excluded due to excessive noise during the collection of psychophysiological data.

The sample of 88 students (20 (22.7%) male and 68 (77.3%) female) comprised 10 males and 37 females in the control group and 10 males and 31 females in the experimental group. The age of the students was between 18 and 47 years old, and mean age ± standard deviation (SD) was 21.28 ± 3.62. No difference was found between groups (*p* = 0.274). The population of higher-education students of health in Portugal in general is largely composed of women. The sample includes more women than men, reflecting the reality of the student population of this institution (according to its internal information management system, the School of Health had 2869 enrolled students, divided between 76.2% women and 23.8% men).

As the inclusion criteria for the study, participants had to be health students from the School of Health—Polytechnic of Porto and be 18 years old or older. In addition, exclusion criteria included having health issues that could compromise the virtual reality experience, such as epilepsy and labyrinthitis.

### 2.3. Procedures

The study was conducted in accordance with the Declaration of Helsinki and received approval from the Ethics Committee of the School of Health—Polytechnic of Porto (CE-ESS-E0094,). All participants were informed and agreed to the terms under which the study was conducted, providing their informed consent to participate.

### 2.4. Instrumentation

Two similar virtual reality immersive environments were designed and developed, using different technologies (graphical computational and 360° video) to provide the participant with an immersive experience simulating the symptoms of schizophrenia. The Virtual Doppelganger Questionnaire [48] was applied to participants before and after the experience. During exposure to the immersive environment, heart rate and galvanic skin response were recorded. After exposure, the Igroup Presence Questionnaire was applied to participants [49].

To provide participants in the two study groups with the same conditions, the immersive environments (graphical computational virtual reality and 360° video) were viewed in the same room and with the same VR equipment. This way, all participants were guaranteed the same sound and image quality. For the immersive experience, HTC Vive Pro™ VR headsets were used, connected to a computer with high processing capacity (Table 1).

The acquisition and analysis system Biopac™ MP160 (Table 2) was used to capture biological signals (cardiovascular changes and galvanic skin response) in a non-invasive way and without disturbing the sensation of immersion, maintaining the freedom of movement of the participant, since they can transmit data wirelessly, which avoids the discomfort and inconvenience of cable connection [50].

All statistical analyses were performed using IBM SPSS Statistics 28. Descriptive statistics, including sociodemographic profiles, absolute and relative frequencies, means, and standard deviations, were computed for the sample. Physiological data were summarized by calculating the average, maximum, and minimum values for each variable over the 7 min exposure period. The effects of intra-group and inter-group variations were analyzed by comparing changes in latent variables over time within each group using paired *t*-tests, and between groups using independent *t*-tests. Differences between the control and experimental groups were assessed using an independent-sample *t*-test. Changes from pre-test (Moment 1) to post-test (Moment 2) within each group were evaluated using a paired-sample *t*-test. Given the adequate sample sizes, normality was assumed based on the central limit theorem [51]. A significance level of 5% was used for all statistical tests.

#### 2.4.1. Immersive Environments

The simulation was designed, based on extensive reports of people with schizophrenia, to help future health professionals understand patients with schizophrenia, allowing them to experience similar symptoms. The experiment aims to determine the influence of exposure to immersive environments on empathy, social distance and attitudes towards people with schizophrenia.

To make the two environments as similar as possible, the room where the 360° video was filmed was reproduced by the virtual reality graphical computational environment. However, the environments were built to be similar, so that, according to the objectives of the work, the results of exposure to the two environments could be compared. The same narrative, the same visual and sound stimuli, as well as the degree of interactivity were used in both environments.

**Figure 1:**
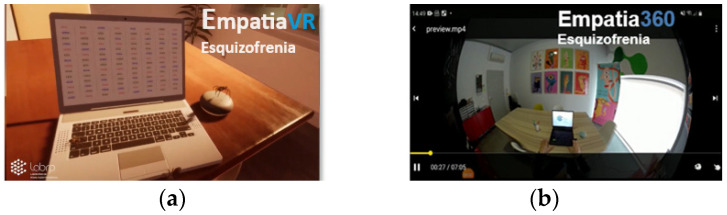
Immersive environments scenarios simulating symptoms of schizophrenia: (**a**) graphical computational virtual reality environment; (**b**) 360-degree video virtual reality environment.

The same scenario was used in both environments (Figure 1). The only difference between the two environments was the technology used for their development. The 360° video images were captured in a closed room, isolated from external stimuli, so that the user could focus on the experience. The graphical computational virtual reality environment reproduced the same room where the 360-degree video images were captured, so that the two immersive environments were as similar as possible.

#### 2.4.2. Immersive Narrative

The immersive narrative simulated an activity where the participant had to perform the Stroop neuropsychological screening test [52]. This test is widely utilized for both experimental and clinical purposes, but in this case, it was solely employed as a task requiring high levels of concentration. The results of the Stroop test were not analyzed, as it was not the focus of this study. The utilization of this test was exclusively intended for participants to undertake a task demanding considerable concentration for a specified duration (approximately 6 min).

At the beginning of the exhibition, the participant had some time to adapt to the environment and received a welcome message from the announcer. Then, the explanation of the task to be carried out was given. In the reading training and color-naming phase, the participant was asked to read the colors and then name them. In the word-reading phase, the participant was asked to read the words aloud regardless of the color in which they were written. In the color-naming phase, the participant was asked to identify the color of the word. At the end, the announcer inquired if the test went smoothly and offers assistance with recovery.

During the exposure, while the participant performed the tasks assigned to them, the immersive environment sent visual and auditory stimuli simulating the hallucinations characteristic of schizophrenia. During exposure, stimuli gradually increased in quantity, intensity, duration, and variability.

The sound stimuli included the following:

- The announcer’s voice indicating and explaining the tasks to be carried out.
- Voices that suggested the participant perform certain actions or discouraged them from the task they were performing.
- Simulated heartbeat and breathing, with sound and intensity gradually increasing throughout the experience.
- Noise from television not tuned to any channel.

The visual stimuli included the following:

- Short periods of blurred vision.
- Movement of the images of the paintings on the wall.
- Glare from windows, lamps, and objects.
- TV screen that turns on and off by itself.
- Spiders that came out from behind the computer and moved across the table towards the participant.

The same visual and sound stimuli occurred with the same duration, intensity and at the same time in both developed environments (graphical computational and 360° video).

#### 2.4.3. The Virtual Doppelganger Questionnaire

The Virtual Doppelganger Questionnaire [48] was applied to participants to measure the effects of a virtual reality simulator on perceptions of schizophrenia. The four dimensions of this questionnaire were used. The dimensions “Empathic feelings for people suffering from schizophrenia”, the “Social distance scale” and “Attitudes toward people with schizophrenia” were tested before and after exposure to the immersive environment. The dimension “Simulation evaluation” was tested after exposure to the immersive environment.

- The dependent measure of “Empathic feelings for people suffering from schizophrenia” uses an index of 12 items. Participants had to rate how well a series of adjectives described their emotions toward people suffering from schizophrenia (rated from 1 = “Not at all”, to 7 = “Extremely”). This measure is related to how the participant felt when thinking about people with schizophrenia (people who hear voices and have delusions that are false ideas or beliefs, even when there is evidence that they are not real). A higher score reflects greater empathy.
- The dependent measure of “The social distance scale” uses an index of 7 items (rated from 0 = “Definitely unwilling” to 3 = “Definitely willing”) and refers to the participant’s interaction with a person with schizophrenia. A decrease in the score reflects a decrease in social distance, suggesting an enhanced interaction with individuals diagnosed with schizophrenia.
- The dependent measure “Attitudes toward people with schizophrenia” uses an index of 7 items (rated in a 9-point scale from 1 = “Strongly disagree” to 9 = “Strongly agree”) and was applied to assess participants attitudes toward people with schizophrenia. A higher score indicates a more positive attitude.
- The simulation evaluation uses an index of 5 items (rated from 9-point scale from 1 = “Strongly disagree” to 7 = “Strongly agree”). This measure was applied after exposure and is related to the reactions of the participant to simulation. A higher score indicates a more positive reaction to the simulation environment.

#### 2.4.4. The Igroup Presence Questionnaire

The Igroup Presence Questionnaire (IPQ), initially developed by Schubert [53], measures mainly the same scales of other instruments like the Slater–Usoh–Steed Presence Questionnaire (SUS) developed by Slater [54] and the Presence Questionnaire (PQ) created by [55]. In its final format, the IPQ allows the quantification of three subscales: spatial presence, realness, and involvement [49]. According to [49], this questionnaire appears to be the only one among the described instruments that has undergone confirmatory factor analysis (CFA).

This study employed three dimensions of the Portuguese version of the Igroup Presence Questionnaire (IPQ) with G1. In this version, validated by [49], the authors reported excellent psychometric properties and supported the three-dimensionality of the scale as theoretically proposed. Different dimensions were used to measure spatial presence (5 items: SP1, SP2, SP3, SP4, SP5), involvement (4 items: INV1, INV2, INV3, INV4), realism (4 items: REAL1, REAL2, REAL3, REAL4) and global presence (1 item: G1). All questions were presented in a five-point Likert scale format utilizing distinct anchors.

- The dependent measure of “global presence” uses an index of 1 item (the item G1 was rated from 1 = “not at all” to 5 = “very much”). This measure is related to the feeling of global presence. A higher score indicates a stronger feeling of presence within the virtual environment.
- The dependent measure of “spatial presence” uses an index of 5 items, (the items SP1, SP2, SP4 and SP5 were rated from 1 = “fully disagree” to 5 = “fully agree”; the item SP3 was rated from 1 = “did not feel” to 5 = “felt present”). This measure pertains to the participant’s sensation of physical presence within the virtual environment. In the items SP1, SP4 and SP5, a higher score indicates a stronger sense of presence within the virtual environment; in the items SP2 and SP3, a lower higher score indicates a stronger sense of presence within the virtual environment. The calculation of this component is performed as follows:
  spatial presence = ((SP1 + SP4 + SP5 + G1) + (−1 × SP2 + 6) + (−1 × SP3 + 6))/6
  After this calculation, a higher score indicates greater spatial presence.
- The dependent measure of “involvement” uses an index of 4 items, (the item INV1 was rated from 1 = “extremely aware” to 5 = “not aware at all”; the items INV2, INV3 and INV4 were rated from 1 = “fully disagree” to 5 = “fully agree”). This measure pertains to the participant’s attention paid to the virtual environment and experienced engagement. In the items INV2 and INV4, a higher score indicates a greater engagement with the immersive environment, in the items INV1 and INV3 a lower score indicates a greater engagement with the immersive environment. The calculation of this component is performed as follows:
  involvement = ((INV2 + INV4) + (−1 × INV1 + 6) + (−1 × INV3 + 6))/4
  After this calculation, a higher score indicates greater involvement.
- The dependent measure of “realism” uses an index of 4 items (the item REAL1 was rated from 1 = “completely real” to 5 = “not real at all”; the item REAL2 was rated from 1 = “not consistent” to 5 = “very consistent”; the item REAL3 was rated from 1 = “about as real as an imagined world” to 5 = “indistinguishable from the real world”; the item REAL4 was rated from 1 = “fully disagree” to 5 = “fully agree”). This measure pertains to the participant’s subjective experience of realism encountered in the virtual environment. A higher score indicates a greater realism of the immersive environment. The calculation of this component is performed as follows:
  realism = (REAL1 + REAL2 + REAL3 + REAL4)/4

#### 2.4.5. Psychophysiological Data

To gather psychophysiological parameters, the Biopac Student Lab System MP36 equipment was employed. This equipment was connected to a computer with two channels: one for ECG and another for EDA. Heart rate (HR) signals and electrodermal activity (EDA) were analyzed using AcqKnowledge (ACK100W) software, followed by extraction and subsequent statistical analysis using IBM SPSS Statistics 28 [56,57]. These data were recorded in both groups (virtual reality group and 360° video). To measure the heart rate (ECG), three vinyl electrodes were positioned on the right shoulder, left shoulder, and sternum. To measure the electrodermal activity (EDA) signal, two electrodes were positioned on the participants, one on the index finger and one on the thumb of the palmar region of the left hand.

## 3. Results

The study results, gleaned from data analysis and interpretation, are presented in the following paragraphs. First, regarding the Virtual Doppelganger Questionnaire, the results of the observed dimensions will be presented: empathic feelings towards individuals with schizophrenia, social distance, attitudes towards individuals with schizophrenia, and simulation evaluation. Next, the results of the application of The Igroup Presence Questionnaire will be presented. Finally, the results of collecting physiological data from participants during exposure to immersive environments will be presented, which included heart rate and electrodermal activity.

### 3.1. Virtual Doppelganger Questionnaire

The values that reflect the reliability of the Virtual Doppelganger Questionnaire application in this study are described in Table 3. Internal consistency assesses the reliability of measurement instruments by ensuring that all items consistently measure the same construct. High internal consistency indicates a reliable instrument, providing confidence in the findings.

The Cronbach’s alpha coefficient reached 0.718 for attitudes toward people with schizophrenia dimension, and 0.735 for empathic feelings for people suffering from schizophrenia dimension, indicating moderate and moderate to good internal consistency/reliability, respectively [58].

For the social distance dimension, the Cronbach’s alpha coefficient reached 0.870; this measurement exhibits strong internal consistency and high reliability [58].

The Cronbach’s alpha coefficient reached 0.534 for simulation evaluation. According to Groth-Marnat [58], this score suggests a low level of internal consistency and reliability.

The Virtual Doppelganger Questionnaire was applied to both groups at two different times, before and after exposure to immersive environments. Table 4 presents the intrasubject differences.

Concerning the difference between moment 1 (M1) and moment 2 (M2) in each of the groups regarding empathic feelings, significant differences were only recorded in the 360° video group (*p* = 0.013, d = 8.73). The same applies to social distance (*p* = 0.027, d = 2.13).

With regard to attitudes, significant differences were observed in both groups, virtual reality (*p* = 0.038, d = 5.23) and 360° video (*p* = 0.044, d = 4.74).

When considering the differences of differences, namely the interaction between the groups, no significant differences were found (Table 5).

When evaluating the simulation, no significant differences were found between the two groups, i.e., control and non-control (Table 6).

### 3.2. The Igroup Presence Questionnaire

The Igroup Presence Questionnaire (IPQ) is regarded as one of the standard tools for measuring presence in virtual environments and has been utilized in numerous research studies [49].

With a Cronbach’s alpha of 0.825 in the Igroup Presence Questionnaire, this study demonstrates strong internal consistency and high reliability (Table 7).

Significant differences were found between the groups regarding involvement (*t* = 2.01; *p* = 0.024; d = 0.56). In the remaining dimensions, although the virtual reality group presented higher scores in the sample, the differences did not prove to be significant.

### 3.3. Physiological Data

During exposure to the immersive environments, physiological data collected from participants included heart rate and electrodermal activity. The collection results are presented in Table 8.

The virtual reality group presents a significantly higher average result (*p* = 0.024) in HR values. The 360° video group presents a significantly higher average result (*p* = 0.024) in EDA peaks values. In the remaining variables, no significant differences were found between the groups.

## 4. Discussion

In this study, two comparable virtual reality immersive environments were created and developed using different technologies (graphical computation and 360° video) to provide participants with an immersive experience that simulates the symptoms of schizophrenia. The Virtual Doppelganger Questionnaire and Igroup Presence Questionnaire were applied to participants. While participants were exposed to the immersive environment, their heart rate and galvanic skin response were monitored.

### 4.1. Virtual Doppelganger Questionnaire

The goal of this study was to examine the impact of exposure to schizophrenia-like experiences about empathy, social distance, and attitudes towards people with schizophrenia using two distinct types of immersive environments, graphical computational virtual reality and 360° video.

The dependent measure of “Empathic feelings for people suffering from schizophrenia” uses an index of 12 items. The Cronbach’s alpha coefficient for the empathy dimension reached 0.735, indicating moderate to good reliability, as it surpassed the threshold of 0.70. This coefficient suggests that the items within the empathy dimension were reasonably correlated, reflecting a coherent construct. Thus, the dimension demonstrates satisfactory internal consistency and moderate reliability [58].

The dependent measure of “The social distance scale” uses an index of 7 items. An alpha coefficient of 0.87 in the social distance dimension indicates a significant correlation among the items within the dimension, highlighting a robust and coherent evaluation of the construct [58].

Although there was an increase in values for both environments, only the 360° video group showed significant improvement in the “Empathetic feelings” and “Social distance” dimensions.

The dependent measure “Attitudes toward people with schizophrenia” uses an index of 7 items. A Cronbach’s alpha of 0.718 in the attitudes dimension suggested a reasonable level of reliability in assessing attitudes [58]. Both immersive environments significantly improved attitudes, and the difference in improvement between the groups was not significant. There were intra-group differences but not inter-group differences.

Simulation evaluation uses an index of 5 items. The alpha coefficient increased to 0.534. This coefficient suggests weak correlations among the items in this dimension, potentially undermining the measurement’s accuracy and validity. Hence, it might be advisable to review and enhance this dimension’s items to bolster its reliability and effectiveness in assessing simulations. This discovery highlights the critical need for thorough evaluation and refinement of measurement instruments to uphold their validity and reliability in psychological assessment settings [58].

Consequently, it may be prudent to reassess and refine the items within this dimension to improve its reliability and effectiveness in evaluating simulations. This finding underscores the importance of rigorously evaluating and refining measurement instruments to ensure their validity and reliability in psychological assessment contexts. However, considering the values obtained, no significant differences were found between the 360° video simulation and virtual reality computer graphics.

### 4.2. Igroup Presence Questionnaire

The Igroup Presence Questionnaire was used for measuring presence in both environments. This questionnaire addressed the dependent measure of “global presence”, “spatial presence”, “involvement” and “realism”. A Cronbach’s alpha of 0.825 in the IPQ indicates that the questionnaire items are highly correlated and measure the same underlying construct cohesively. Thus, the measure is considered highly reliable and suitable for assessing group presence in specific contexts.

Significant differences were found between the groups regarding involvement. In the remaining dimensions, although the virtual reality group presented higher scores in the sample, the differences did not prove to be significant. These results may be related to the number of elements in the study; therefore, it is suggested that studies be carried out with larger samples.

### 4.3. Heart Rate Signals and Electrodermal Activity

Exposure to the virtual reality computer graphics environment caused higher HR values, while exposure to the 360° video caused higher EDA peaks values. No significant differences were found between the groups in the remaining variables.

These results require further studies that can analyze the relationship between HR values with the “involvement” dimension and EDA peak values with the “realism” dimension, values presented in the IPQ.

### 4.4. Limitations

In the simulation evaluation, the alpha coefficient of 0.534 suggests a weak correlation among the items in this dimension. Consequently, it may be prudent to reassess and refine the items within this dimension to improve its reliability and effectiveness in evaluating simulations. This finding underscores the importance of rigorously evaluating and refining measurement instruments to ensure their validity and reliability in psychological assessment contexts.

### 4.5. Future Research

As future research, it is suggested to apply the study in different contexts to promote health literacy about schizophrenia, such as health professionals, family members, friends and co-workers who deal with people with schizophrenia in their daily lives.

Another interesting aspect would be the development of a similar immersive environment in augmented reality to compare the impact with virtual reality computer graphics and 360° video.

## 5. Conclusions

The creation of immersive environments is complex and requires substantial human and technological resources. Cost and development time are two of the main challenges to consider as well as some reluctance to use new technology. Virtual reality computer graphics and 360° video are different technologies that allow the creation of this type of environment. Each of these technologies has its advantages and limitations. Overall, 360° video entails lower costs and less development time; on the other hand, its limitations are associated with less interactivity and the use of physical world scenarios. Virtual reality computer graphics allows the creation of more interactive, flexible and dynamic environments; however, it requires more development time, more specialized work and consequently greater costs.

A more effective choice of the appropriate technology to respond to a given problem results in a more appropriate solution and increased response capacity to a greater number of requests that may be made.

The development of immersive environments to promote health literacy, empathy and combat stigma towards mental illness must encompass three important dimensions, “Empathic feelings”, “Social distance” and “Attitudes”. The selection of the appropriate technology must consider the intended purpose.

VR computer graphics environments can provide a more interactive and personalized experience but can be more expensive and require more hardware resources. Although more accessible, less complex and faster to produce, 360° video allows less interactivity, less flexibility, less personalization and less control over the experience.

This study established that, since no significant differences were found between these two types of immersive environments in terms of their impact on the dimensions under study, the choice between them should depend on the specific objectives of the project, the needs of the target audience, and the resources available. More tailored environments might be more effective for certain outcomes, suggesting that customization could play a crucial role. Additionally, the limited sample size in this study may have influenced the findings, indicating a need for further research with a larger and more diverse population. Furthermore, the effectiveness of the technology might require repeated use rather than a single exposure, as ongoing engagement could enhance its impact. Therefore, the decision should not be based on the type of immersive environment itself, but on the functionalities required to achieve a certain result, considering these factors.

## Figures and Tables

**Table 1:** Equipment used in exposure to immersive environments (graphical computational virtual reality and 360° video).

Equipment	Specifications
Computer	CPU: Intel^®^ Core™ i7-9700 K (3.60 GHz–4.90 GHz) Graphics Card: NVIDIA^®^ GeForce^®^ RTX 2080 Ti Memory: 64 GB RAM
VR Headset HTC Vive Pro™	High-resolution Dual AMOLED 3.5 diagonal screens 1440 × 1600 pixels per eye (2880 × 1600 pixels combined) Refresh rate: 90 Hz Field of view: 110 degrees Integrated microphones with 3D Spatial Audio Four SteamVR Base Station 2.0: 10 m × 10 m VIVE Wireless Adapter

**Table 2:** Equipment used in exposure to immersive environments (graphical computational virtual reality and 360° video) for physiological data collection (ECG and galvanic skin response).

Equipment	Specifications
ECG	Number of Channels: 16 Absolute Maximum Input: ±15 V Operational Input Voltage: ±10 V A/D Resolution: 16 Bits Accuracy (% of FSR): ±0.003 Input Impedance: 1.0 MΩ Amplifier Module Isolation: Provided by the MP unit, isolated clean power CE Marking: EC Low Voltage and EMC Directives Leakage Current: <8 µA (Normal), <400 µA (Single Fault) Fuse: 2 A (fast blow). Memory: 64 GB RAM
Galvanic skin response	Signal Type: PPG plus EDA Resolution: PPG: FSR/4096, (4.88 mV); EDA: 0.012 μS (min step) Operational Range: 10 m Transmitter: Ultra-low power, 2.4 GHz bi-directional digital RF transmitter; Rate: 2.000 Hz (between transmitter and receiver)

**Table 3:** Internal consistency for the dimensions of the Virtual Doppelganger Questionnaire.

Dimension	Items	Cronbach’s Alpha
Empathic feelings for people suffering from schizophrenia	12	0.735
Social distance	7	0.870
Attitudes toward people with schizophrenia	7	0.718
Simulation evaluation	5	0.534

**Table 4:** The Virtual Doppelganger Questionnaire intrasubject differences.

Dimension	Immersive Environment	Mean Difference M2-M1	sd	*t*	*p*	Cohen’s D
Empathic feelings	Virtual reality	0.81	1.31	0.62	0.270	
	360° video	3.15	1.36	2.31	0.013	8.73
Social distance	Virtual reality	0.02	0.27	0.08	0.469	
	360° video	−0.66	0.33	−1.98	0.027	2.13
Attitudes	Virtual reality	1.38	0.76	1.81	0.038	5.23
	360° video	1.29	0.73	1.75	0.044	4.74

sd = standard deviation; *t* = *t*-statistic; *p* = *p*-value.

**Table 5:** The Virtual Doppelganger interaction.

Dimension	Immersive Environment	Mean Difference	sd	*t*	*p*
Empathic feelings	Virtual reality	−2.34	1.89	−1.23	0.110
	360° video				
Social distance	Virtual reality	0.68	0.42	1.61	0.056
	360° video				
Attitudes	Virtual reality	0.09	1.07	0.08	0.466
	360° video				

sd = standard deviation; *t* = *t*-statistic; *p* = *p*-value.

**Table 6:** The Virtual Doppelganger simulation evaluation.

Dimension	Immersive Environment	Mean	sd	*t*	*p*
Simulation evaluation	Virtual reality	34.47	2.34	0.42	0.338
	360° video	33.27	2.11		

sd = standard deviation; *t* = *t*-statistic; *p* = *p*-value.

**Table 7:** Igroup Presence Questionnaire.

Dimension	Immersive Environment	Mean	sd	*t*	*p*	Cohen’s D
Spatial presence	Virtual reality	3.84	0.44	0.42	0.338	
	360° video	3.59	0.67			
Involvement	Virtual reality	3.67	0.71	2.01	0.024	0.56
	360° video	3.61	0.50			
Realism	Virtual reality	2.95	0.57	0.47	0.32	
	360° video	2.75	0.63			
Global presence	Virtual reality	3.48	0.44	1.56	0.061	
	360° video	3.32	0.47			

sd = standard deviation; *t* = *t*-statistic; *p* = *p*-value.

**Table 8:** Heart rate (HR) signals and electrodermal activity (EDA).

Physiological Data	Immersive Environment	N	Average	sd	Coefficient of Variation	*p*
HR average	Virtual reality	47	98.18	14.84	0.15	0.024
	360° video	41	89.83	19.24	0.21	
HR maximum	Virtual reality	47	123.08	17.43	0.14	0.160
	360° video	41	116.99	22.80	0.19	
HR minimum	Virtual reality	47	73.48	14.38	0.20	0.239
	360° video	41	69.53	16.86	0.24	
EDA peaks	Virtual reality	47	57.66	24.95	0.43	0.024
	360° video	41	68.49	17.98	0.26	
EDA amplitude	Virtual reality	47	0.50	0.30	0.61	0.143
	360° video	41	0.40	0.29	0.71	

sd = standard deviation; *p* = *p*-value.

## Data Availability

The raw data supporting the conclusions of this article will be made available by the authors on request.
